# Contribution of autophagy to ocular hypertension and neurodegeneration in the DBA/2J spontaneous glaucoma mouse model

**DOI:** 10.1038/s41420-018-0077-y

**Published:** 2018-07-17

**Authors:** Joshua Hirt, Kris Porter, Angela Dixon, Stuart McKinnon, Paloma B. Liton

**Affiliations:** Duke University, Department of Ophthalmology, Durham, NC USA

**Keywords:** Glaucoma, Autophagy

## Abstract

Glaucoma is a progressive optic neuropathy characterized by axonal degeneration and retinal ganglion cells loss. Several factors have been postulated to play a role in glaucoma, elevated intraocular pressure (IOP) being the best well-known causative factor. The mechanisms leading to ocular hypertension and glaucoma are still not fully understood. An increasing number of evidence indicates a role of autophagy in the pathophysiological process of ocular hypertension and glaucoma. However, while all of the studies agree that autophagy is induced in RGCs in response to injury, autophagy was found to either protect or promote cell death depending on the experimental model used. In order to gain more insight into both, the role of autophagy in the pathogenesis of glaucoma and the effect of chronic IOP elevation in the autophagy pathway, we have investigated here for the first time autophagy in the iridocorneal angle region, retinal ganglion cell bodies, and ON axons in the spontaneous ocular hypertensive DBA/2J mouse glaucoma model and in the transgenic DBA/2J::GFP-LC3 mice, generated in our laboratory. Our results indicate decreased autophagic flux in the outflow pathway cells in the DBA/2J mice, characterized by increased levels of LC3-II and p62 together with a decrease in the lysosomal marker LAMP1, evaluated by western blot and immunofluorescence. Elevated presence of autophagic vacuoles in the DBA/2J and, in particular, in the DBA/2J::GFP-LC3 mice was also observed. Expression of the GFP-LC3 transgene was associated to higher cumulative IOP in the DBA/2J background. In addition to higher elevation in IOP, DBA/2J::GFP-LC3 were characterized by further RGCs and exacerbated axonal degeneration compared to DBA/2J. This was accompanied by the notable high presence of autophagic figures within degenerating axons. These results strongly suggest overactivation of autophagy as a potential cellular mechanism leading to ON degeneration in the chronic hypertensive DBA/2J mice.

## Introduction

Glaucoma is a progressive optic neuropathy, second leading cause of permanent blindness worldwide. Several factors have been postulated to play a role in glaucoma including ischemia excitotoxicity, neurotrophic insufficiency, and elevated intraocular pressure (IOP). Among them, elevated IOP remains the best-established causative factor^1,2^. Elevated IOP results from the failure of the trabecular meshwork (TM)/Schlemm’s canal (SC) conventional outflow pathway, a tissue located in the anterior segment of the eye, to maintain appropriate levels of aqueous humor (AH) outflow resistance^3^. The exact mechanisms by which elevated IOP triggers axonal degeneration and retinal ganglion cell (RGC) death is currently unknown. Directly, IOP-related mechanical injury to the optic nerve (ON) head at the lamina cribrosa level might lead to ischemia-hypoxia damage, blockage of axonal transport, and growth factor deprivation, which culminate in RGC death^4,5^. Indirectly, damage to RGCs might result from the action of factors released by activated glial cells and astrocytes located in the lamina cribrosa^6^. Regardless of the mechanisms that initiate glaucomatous retinal damage, evidence indicate that these pathways converge on a common cellular event that triggers axonal degeneration and RGC death in glaucoma.

Autophagy is an evolutionarily conserved pathway that mediates cellular degradation through the action of lysosomes. Physiological levels of autophagy are essential for the maintenance of cellular homeostasis, and it is rapidly upregulated during various stress conditions (i.e., oxidative stress, mechanical stress). Dysfunction of the autophagy pathway, especially with aging, often promotes human diseases and several neurodegenerative disorders^7–9^. Paradoxically, despite primarily having a pro-survival role, neural tissue autophagy plays an important role in both neuroprotection as well as neuronal injury and death depending on the physiological and pathological environment^10^. Excessive or uncontrolled levels of autophagy are able to induce autophagic cell death in a caspase-dependent or in a caspase-independent manner, defined by the massive autophagic vacuolization of the cytoplasm^11,12^.

Although not extensively studied, an increasing number of evidence indicates a role of autophagy in the pathophysiological process of ocular hypertension and glaucoma^13–19^. In a recent work, our laboratory reported the dysregulation of autophagy with aging and in TM cells isolated from glaucoma cadaver eyes^17^. A connection between autophagy and neurodegeneration in glaucoma is strongly supported from the discovery that the optineurin and TANK Binding Kinase (TBK1), normal tension glaucoma (NTG)-associated genes, are essential players in the autophagic degradation of damaged mitochondria, a process known as mitophagy^20,21^. Activation of autophagy in RGCs in response to injury (ON transection) or elevated IOP (acute or chronic) has been reported in different induced models of experimental glaucoma^22–28^. While all of the studies agree that autophagy is induced in RGCs in response to injury, autophagy was found to either protect or promote cell death depending on the experimental model used. Piras et al.^28^ observed the occurrence of autophagic retinal cell death following I/R produced by acute IOP increase in rats. In contrast, by using a knockout mouse model of autophagy impairment^29^, Rodriguez-Muela et al.^22^ demonstrated that genetic downregulation of autophagy increases RGC death following ON axotomy, whereas pharmacological upregulation of autophagy with rapamycin reduces RGC loss in vivo. Similar neuroprotective effect of rapamycin was described in the episcleral vein cautery chronic ocular hypertensive model rat model in ref.^26^. Contradictory, inhibition of autophagy with 3-methyladenine also showed protective effect in the same model as in ref.^25^.

In order to gain more insight into both the role of autophagy in the pathogenesis of glaucoma and the effect of chronic IOP elevation in the autophagy pathway, we have investigated here for the first time autophagy in the neuroretina, ON axons, and iridocorneal angle region tissues of the spontaneous ocular hypertensive DBA/2J mouse model of glaucoma. DBA/2J is an inbred strain that progressively develops eye abnormalities that closely mimic human glaucoma. At around 6 months of age, DBA/2J mice experience iris atrophy and pigment dispersion. Accumulation of pigment in the TM obstructs AH drainage leading to high IOP, and subsequent loss of RGCs and ON axons. DBA/2J offer the advantage to share many similarities to human disease, in contrast to inducible experimental models. These include age-related variable progression of ON atrophy in response to elevated IOP and a regional pattern of RGC death and ON excavations. DBA/2J have become the most widely used mouse model to decipher mechanisms of glaucomatous neurodegeneration^30^. Our results indicate decreased autophagic flux in the outflow pathway cells and suggest overactivation of autophagy as a potential cellular mechanism leading to ON degeneration in the chronic hypertensive DBA/2J mice.

## Results

### Generation of DBA/2J::GFP-LC3 mice

**Fig. 1:**
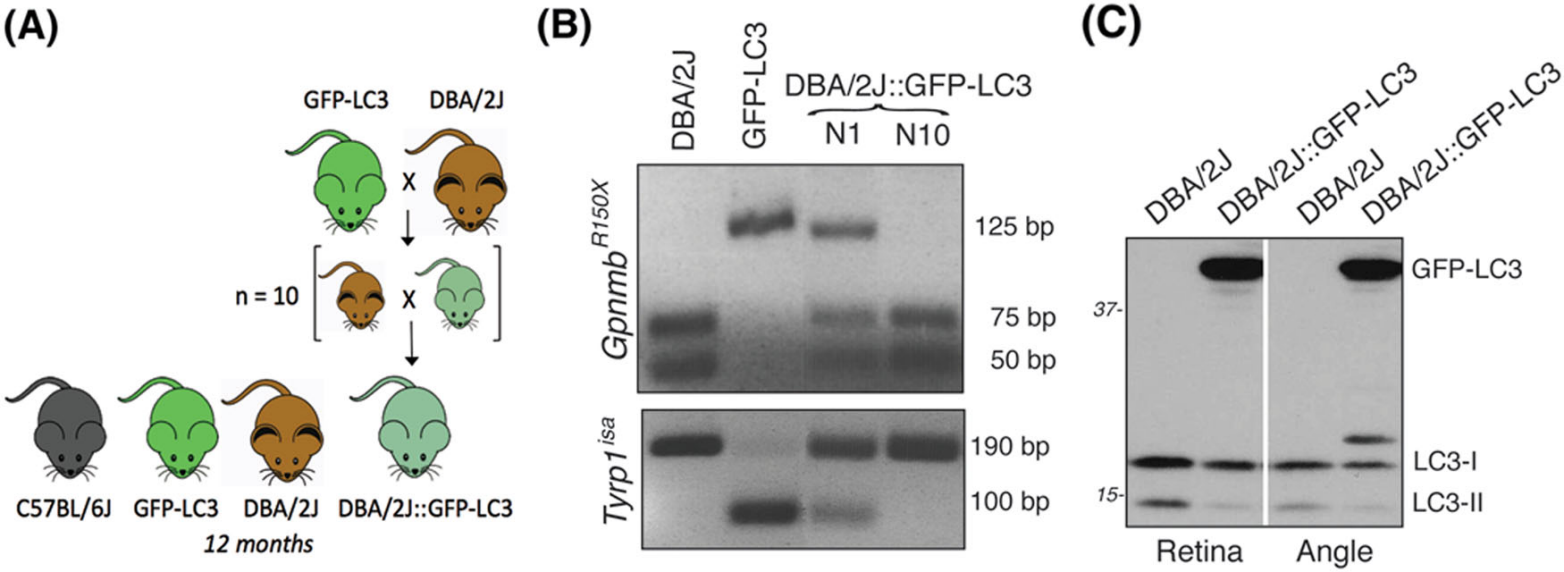
Generation of DBA/2J::GFP-LC3. **a** GFP-LC3 mice were crossbred with DBA/2J mice. The progeny was backcrossed for ten generations into DBA/2J background. Twelve-month-old mice were used for the studies. **b** PCR genotyping analysis showing the presence of the GpnmbR150x and Tyrp1b allele mutations in DBA/2J::GFP-LC3 (N10). **c** Western blot analysis in protein whole retinal and angle region tissue lysates (10 μg) showing the expression of the transgene GFP-LC3 (44 kDa)

To monitor autophagy in the angle and retina tissues of the spontaneous ocular hypertensive glaucoma model DBA/2J mice, we crossbred transgenic GFP-LC3 mice, which ubiquitously express the autophagosome marker LC3 fused to GFP^31^, with DBA/2J mice. LC3-I (16–18 kDa) is a soluble protein that diffuses throughout the cytosol. Upon induction of autophagy, LC3-lyI is lipidated into LC3-II (14–16 kDa) and incorporated within the autophagosome membrane. GFP-LC3 can then be visualized as GFP puncta preferentially located in the perinuclear region. GFP-LC3 mice are widely used for monitoring autophagy in vivo. Progeny was then backcrossed for ten generations into the DBA/2J background to obtain DBA/2J::GFP-LC3 mice (Fig. 1a). The presence of the Gpnmb^R150x^ and Tyrp1^b^ allele mutations were confirmed by genotyping (Fig. 1b), as detailed in Materials and Methods section. Transgene expression in the angle and retina tissues was confirmed by western blotting (WB) (Fig. 1c) and immunofluorescence (Figs. 3 and 6). All parameters were analyzed in 12-month-old animals. As seen in Supplementary Information, no apparent morphological differences were observed in the angle region or retina tissue between the transgenic mice and their respective controls.

### IOP in GFP-LC3 and DBA/2J::GFP-LC3 mice

**Fig. 2:**
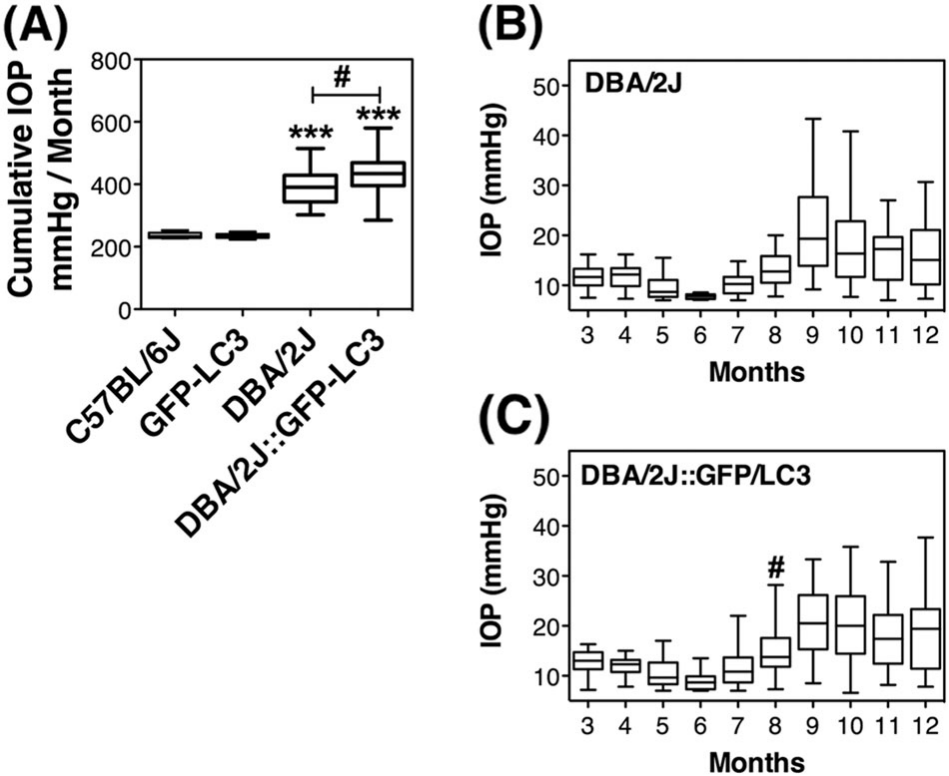
Intraocular pressure in GFP-LC3 and DBA/2J::GFP-LC3 mice. **a** Mean cumulative IOP calculated as mmHg/month. **b** and **c** Mean monthly IOP measurements in DBA/2J and DBA/2J::GFP-LC3 mice, respectively; ^#^*p* < 0.05, ***p < 0.001, Tukey test; *n*_C57BL/6J_ = 18, *n*_DBA/2J_ = 32, *n*_GFP-LC3_ = 22, *n*_DBA/2J::GFP-LC3_ = 40. ^(*)^DBA/2J and DBA/2J::GFP-LC3 compared to C57BL/6J and GFP-LC3, respectively. ^(#)^DBA/2J::GFP-LC3 compared to DB2/2J

GFP-LC3 mice did not show any difference in IOP compared to the C57BL/6J controls (Fig. 2a, 235 ± 7 mmHg/month vs. 237 ± 9 mmHg/month, *n* = 22 and 18, respectively), indicating that the presence of the transgene does not affect outflow pathway tissue function, at least, up to the time tested (12 months old). As expected, a significant elevation in IOP was observed in DBA/2J compared to C57BL/6J mice (391 ± 54 mmHg/month vs. 237 ± 9 mmHg/month, *p* < 0.0001, *n* = 32 and 18, respectively). Elevation in IOP became clearly detectable at 9 months and was sustained through the duration of the study (Fig. 2b). DBA/2J::GFP-LC3 mice also demonstrated significant higher cumulative IOP compared to C57BL/6J and GFP-LC3 mice (430 ± 69 mmHg/month vs. 237 ± 9 mmHg/month and 235 ± 7 mmHg/month, *p* < 0.0001, *n* = 40, 18, and 22). Interestingly, a small magnitude but significant higher elevated IOP in DBA/2J::GFP-LC3 was noticed compared to DBA/2J mice (429 ± 69 vs. 391 ± 54 mmHg/month, *p* = 0.01, *n* = 40 and 32, respectively). When analyzed over time, we observed DBA/2J::GFP-LC3 mice to develop elevated IOP at earlier times (8 months) that was sustained at an average of ∼2 mmHg higher throughout the duration of the study compared to DBA/2J (Fig. 2b, c).

### Autophagy in the angle region of DBA/2J mice

**Fig. 3:**
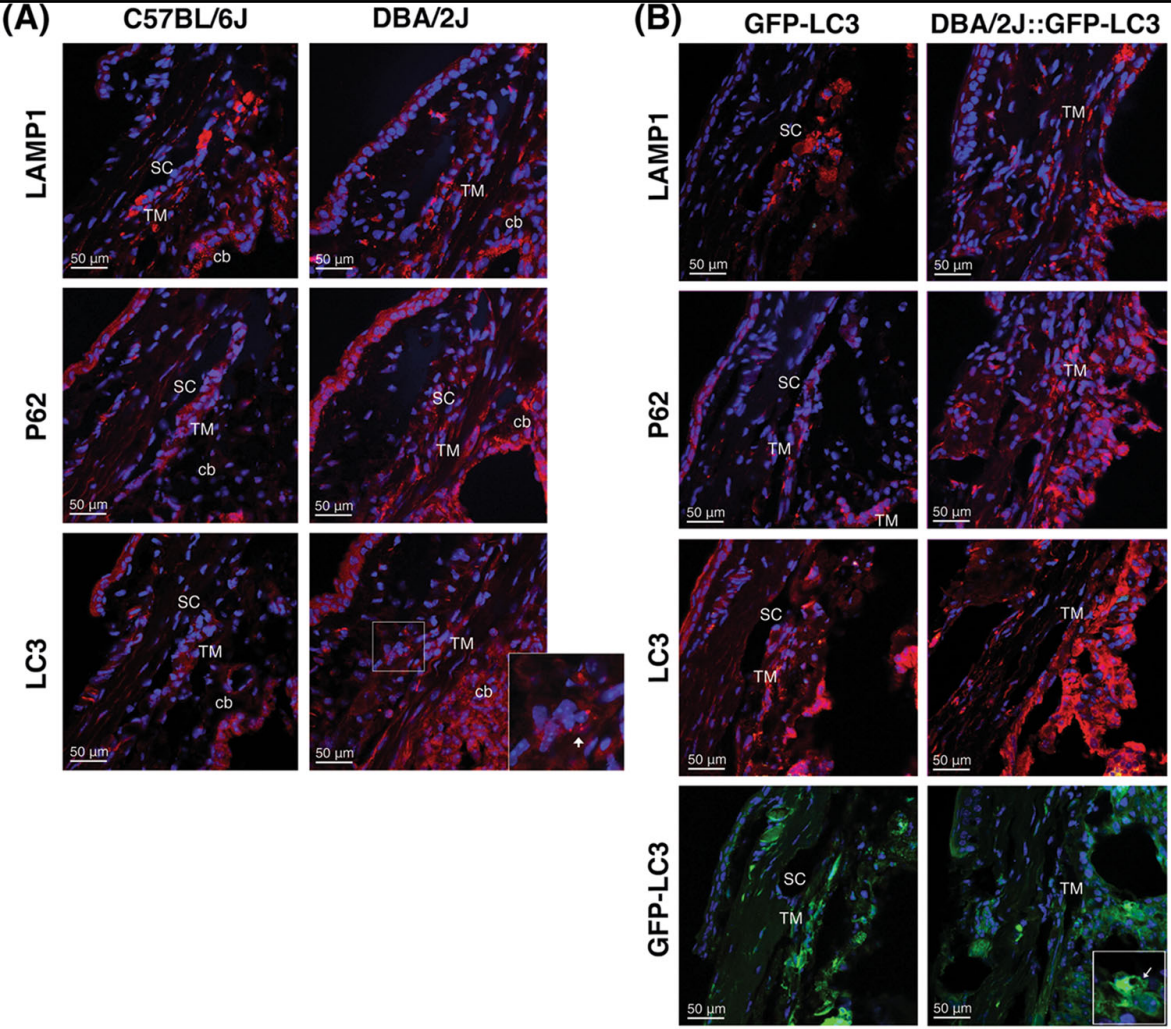
Immunofluorescence staining of autophagy lysosomal markers in the iridocorneal angle region of C57BL/6J and DBA/2J mice. Representative immunofluorescence staining of LAMP1, p62, and LC3 in the angle region of non-GFP (**a**) and GFP-LC3 transgenic mice (**b**). Red: specific antibody staining; blue: DAPI; green: GFP-LC3 fluorescence. SC Schlemm’s canal, TM trabecular meshwork, cb ciliary body. Arrows indicate the presence of LC3 puncta

**Fig. 4:**
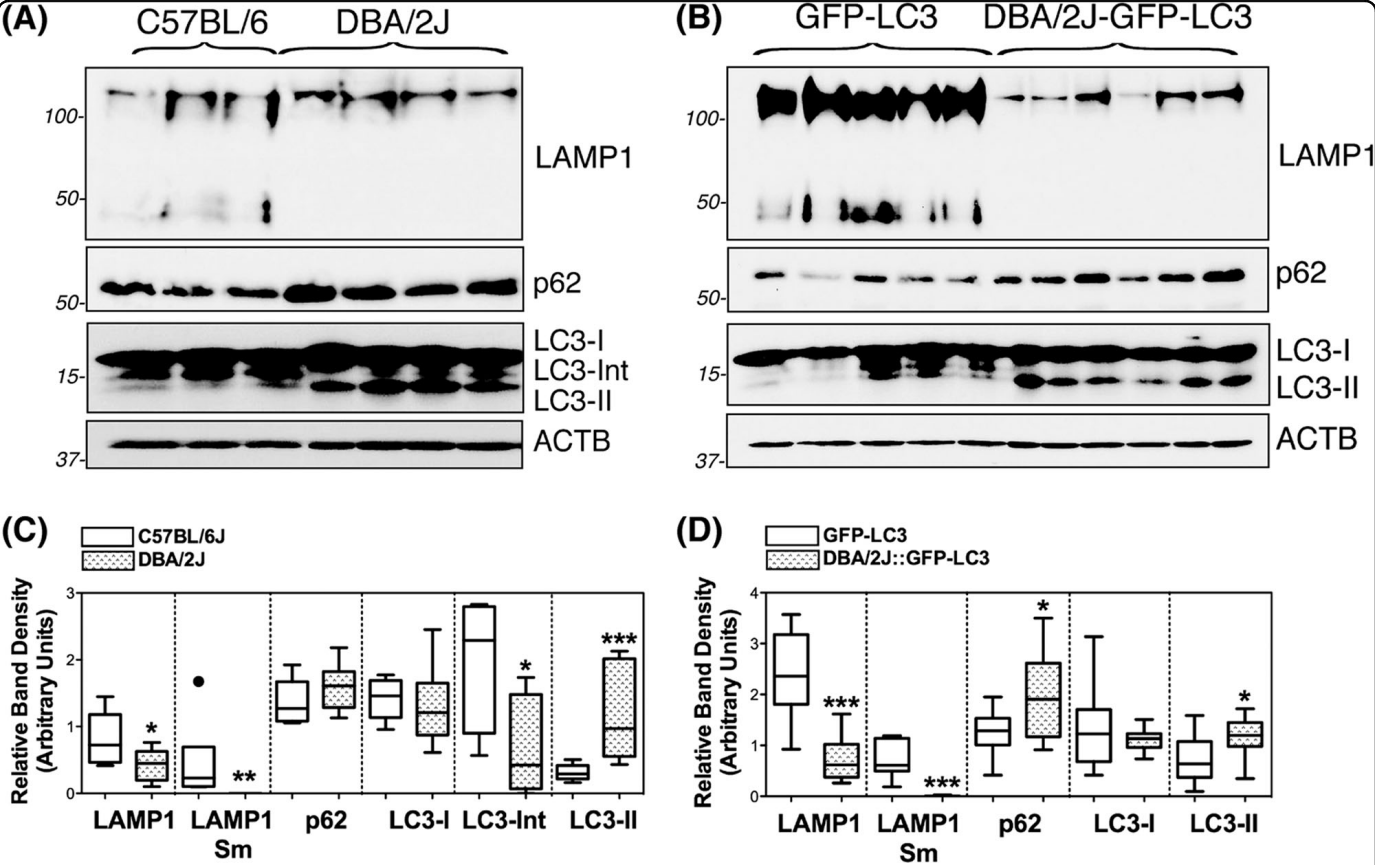
Western-blot analysis of autophagy lysosomal markers in dissected iridocorneal angle regions of C57BL/6J and DBA/2J mice. Protein expression levels of LC3, p62, and LAMP1 in whole-tissue lysates (5–10 μg) of dissected iridocorneal angle region from non-GFP (**a**) and GFP-LC3 transgenic mice (**b**) analyzed by western blot; **c** and **d** represent the normalized relative protein levels calculated from densitometric analysis of the blots. Data are the means ± SD; *p < 0.05, **p < 0.01, ***p < 0.001, Tukey test; *n*_C57BL/6J_ = 6, *n*_DBA/2J_ = 9, *n*_GFP-LC3_ = 10, *n*_DBA/2J::GFP-LC3_ = 10. Sm: small band, non-glycosylated form

**Fig. 5:**
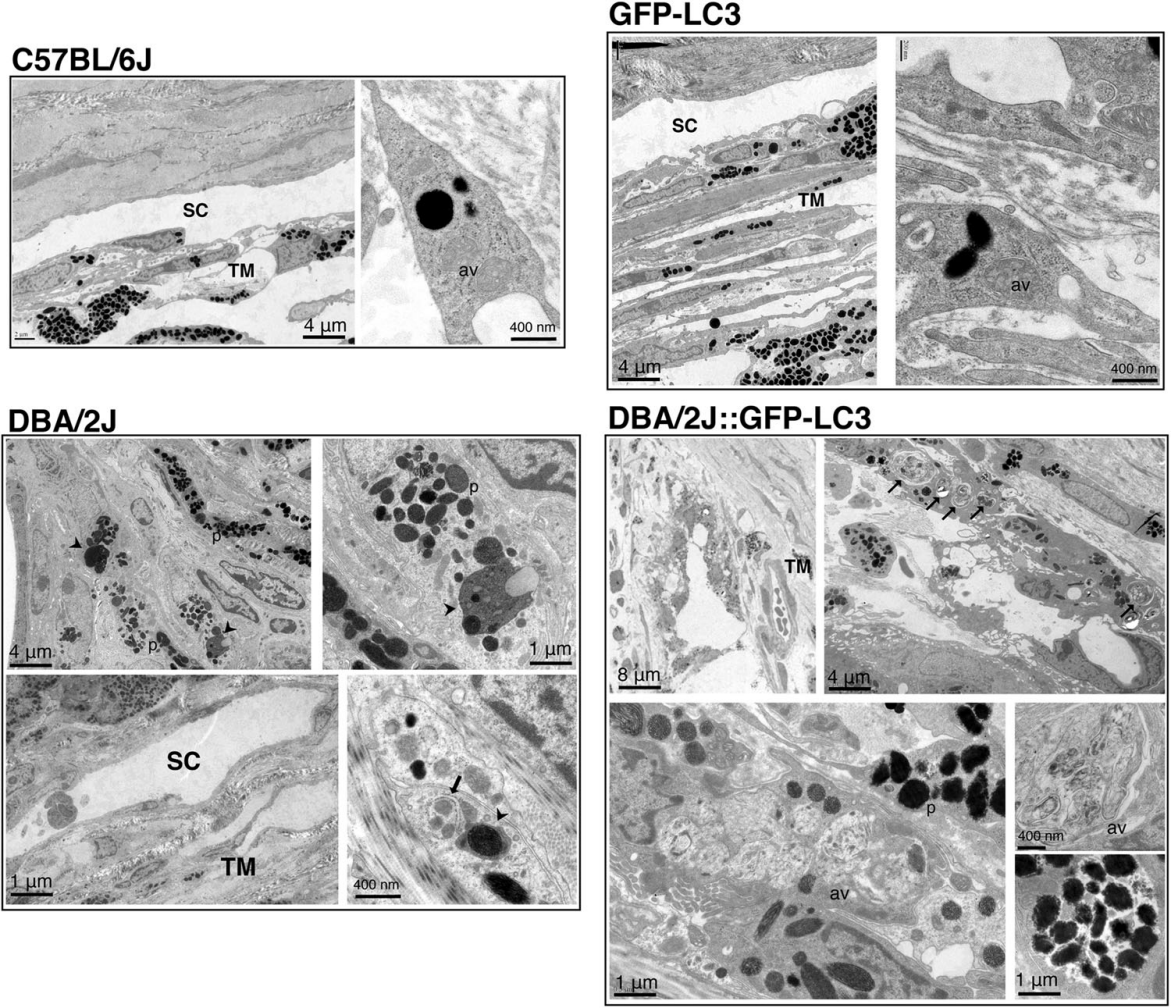
Ultrastructural analysis of autophagy in the outflow pathway tissue of C57BL/6J, GFP-LC3, DBA/2J, and DBA/2J::GFP-LC3. Micrographs are representative of at least six different animals per group. Arrows, av: autophagic vacuoles, p: pigment, arrowheads: pigment-loaded vacuoles

The occurrence of autophagy and autophagic flux in the iridocorneal angle region was investigated by monitoring the protein levels of the autophagosome marker LC3 (I and II), the lysosomal marker LAMP1, and the autophagy receptor p62, both by immunofluorescence (Fig. 3) and WB (Fig. 4). Qualitatively, DBA/2J mice displayed a decreased in intensity staining for LAMP1 specific to the SC/TM region (no difference was observed in the ciliary body tissue) compared to C57BL/6J mice. In contrast, LC3 and p62 staining were relatively more intense in the ciliary body and SC/TM tissues in the DBA/2J mice. Most importantly, both LC3 and p62 exhibited a punctate rather than diffused pattern of staining suggesting the higher presence of autophagosomes in the TM/SC region of DBA/2J mice (Fig. 3a). Similar results, were observed between GFP-LC3 and DBA/2J::GFP-LC3 mice (Fig. 3b). Quantitative WB analysis in protein lysates from dissected angle tissue confirmed the decreased expression of LAMP1 (both the fully glycosylated ∼100 kDa band and the small non-glycosylated ∼50 kb band, Sm) as well as higher protein levels of p62 and LC3-II in DBA/2J and DBA/2J::GFP-LC3 mice compared to their respective C57BL/6J and GFP-LC3 controls (Fig. 4a, b). The LC3 antibody recognized a third band of ∼17.5 kDa running between LC3-I and LC3-II. This unusual LC3 band (LC3-Int) has been suggested to be a processing intermediate of LC3-I. Interestingly, C57BL/6J mice demonstrated significantly higher levels of LC3-Int, which was practically absent in DBA/2J and DBA/2J::GFP-LC3. Electron micrographs showed the presence of isolated autophagic vacuoles (av) within TM cells from C57BL/6J and GFP-LC3 (Fig. 5). Pigment-loaded vacuoles were observed in trabecular cells in DBA/2J, likely resulting from the phagocytic uptake of iris pigment (Fig. 5, arrowheads). The incidence of pigment-loaded and autophagic vacuoles was found to be much higher in DBA/2J::GFP-LC3 (Fig. 5, arrows).

### Autophagy in RGCs of DBA/2J mice

**Fig. 6:**
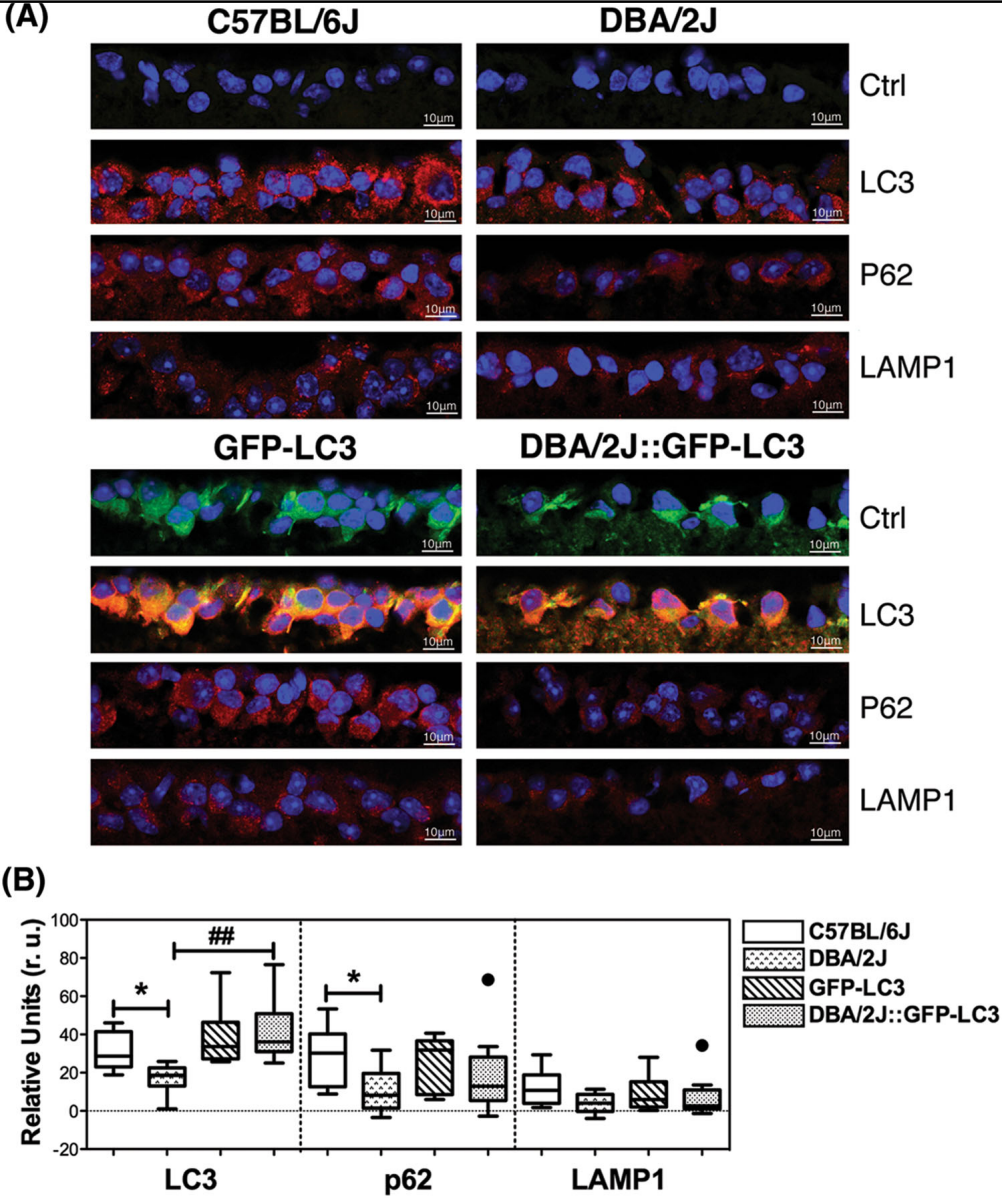
Evaluation of autophagy in the GCL of C57BL/6J and DBA/2J mice. **a** Representative immunofluorescence staining (red) of LAMP1, p62, and LC3 in the GCL of non-GFP and GFP-LC3 transgenic mice. Blue fluorescence represents DAPI nuclear counterstaining. **b** Standardized relative quantification of immunofluorescence staining. Data are the means ± SD; *p < 0.05, ^##^*p* < 0.01, Tukey test; *n*_C57BL/6J_ = 6, *n*_DBA/2J_ = 9, *n*_GFP-LC3_ = 10, *n*_DBA/2J::GFP-LC3_ = 11

**Fig. 7:**
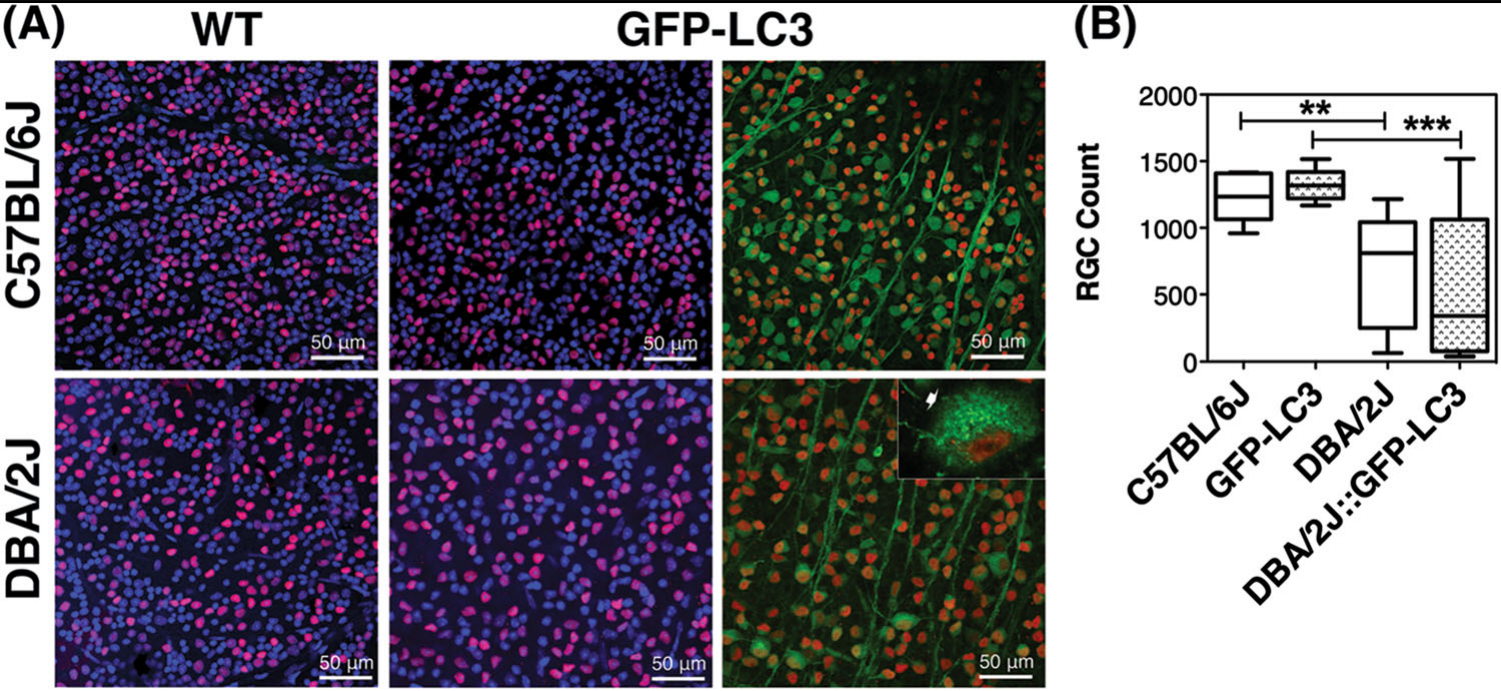
RGC quantification in C57BL/6J and DBA/2J mice. **a** Representative images from whole retinal flat mounts from no-GFP and GFP mice stained with anti-Brn3a (red fluorescence). Green represents endogenous GFP-LC3 fluorescence. Arrow in the inset indicates LC3 puncta. **b** Quantification of the number of Brn3a-positive cells. Data are the means ± SD; **p < 0.01, ^***^p < 0.001, Tukey test; *n*_C57BL/6J_ = 10, *n*_DBA/2J_ = 14, *n*_GFP-LC3_ = 9, *n*_DBA/2J::GFP-LC3_ = 12

Autophagy in the ganglion cell layer of the retina was evaluated by IF. As seen in Fig. 6a, b, DBA/2J mice displayed decreased protein levels of LC3, p62, and LAMP1, although this latter did not reach statistical significance. No major changes besides the anticipated LC3 levels increase in GFP-LC3 and DBA-2J::GFP-LC3 were observed with the expression of the transgene. We additionally investigated the presence of autophagosomes (GFP-LC3 puncta) specifically in RGCs in whole retinal flat mounts using an anti-Brna3 antibody (Fig. 7a). Although LC3 puncta were detected in Brna3a-stained positive cells (RGCs) in DBA/2J::GFP-LC3 mice, they were also seen in GFP-LC3 controls with no striking difference observed among the different groups. Quantification of RGC (Brn-3a-positive cells) showed an anticipated RGC bodies loss in DBA/2J compared to age-matched C57BL/6J mice (Fig. 7b, 690.8 ± 409.9 vs. 1225 ± 178.4 total RGC, *p* = 0.0082, *n*_DBA/2J_ = 12, *n*_C57BL/6J_ = 10). No significant differences in the total number of Brn-3a-positive cells were found between C57BL/6J and GFP-LC3 cells. Although not statistically significant, a trendy 20% decrease in RGC count was also observed in DBA/2J::GFP-LC3 compared to DBA/2J (556.7 ± 543.3 vs. 690.8 ± 409.9 total RGC, *p* = 0.490, *n*_DBA/2J_ = 12, *n*_DBA/2J::GFP-LC3_ = 14).

### Axonal degeneration and autophagy in DBA/2J::GFP-LC3 mice

**Fig. 8:**
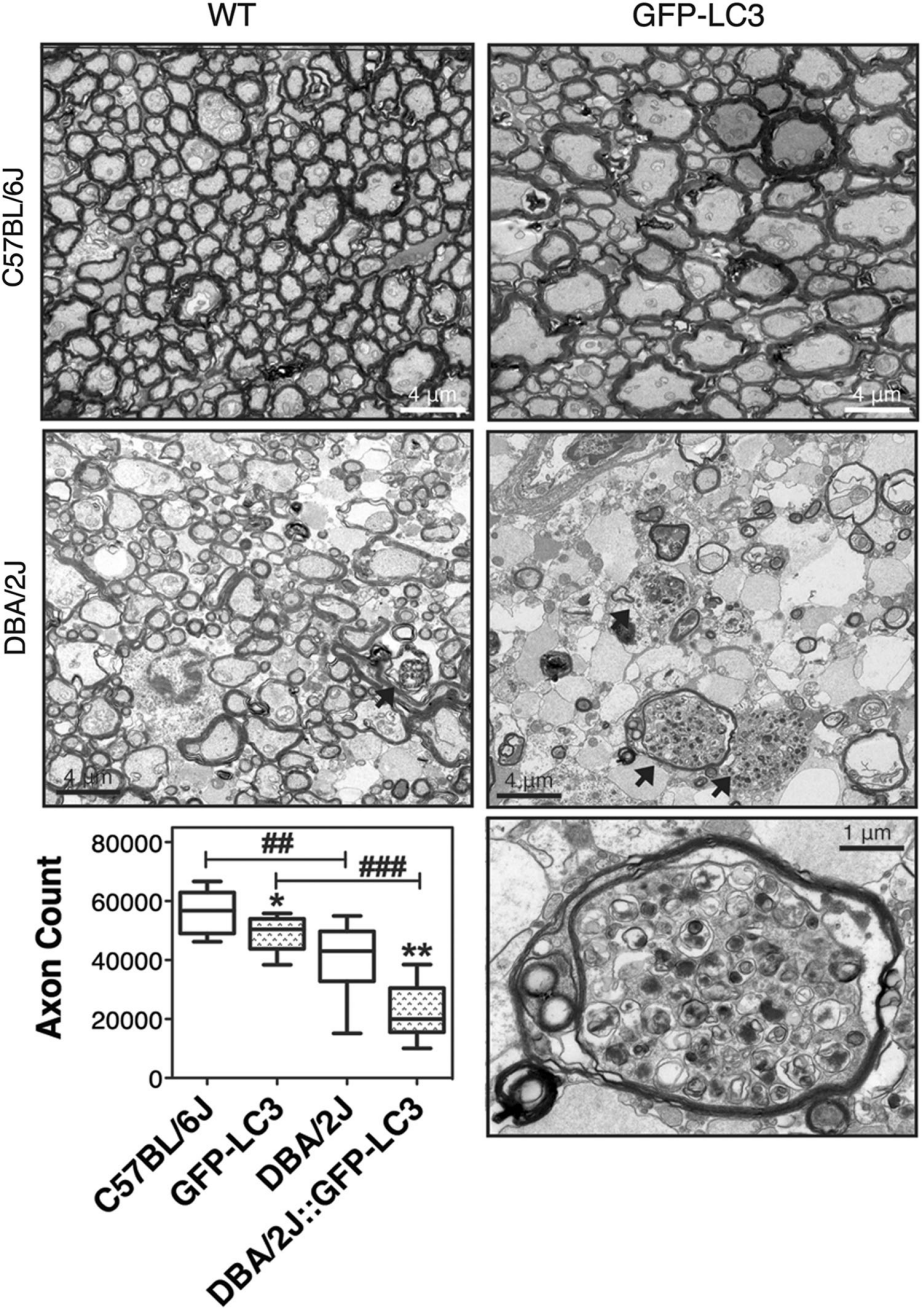
Evaluation of axonal degeneration. Images show representative transmission electron micrographs of cross-sectional areas of the myelinated segment of the ONs in C57BL/6J, DBA/2J, GFP-LC3, and DBA/2J::GFP-LC3 mice. Arrows indicate the presence of autophagic vacuoles. Higher magnification image of an autophagic vacuole in a degenerating axon from DBA/2J::GFP-LC3 mouse is displayed at the bottom left panel. Total axon number counts are plotted in the graph. Data are the means ± SD; *p < 0.05, **, ^##^*p* < 0.01, ^###^*p* < 0.001, Tukey test; *n* = 10

Axonal degeneration was evaluated by determining the number of RGC axons in cross-sectional areas of the myelinated segment of the ONs (Fig. 8). An expected decrease in total axon number was observed in DBA/2J compared to C57BL/6J mice (39,956 ± 13,692 vs. 56,288 ± 7219 axons, *p* = 0.007, *n* = 10). Extensive gliosis and numerous degenerating axon profiles with multi-laminar myelin sheaths could be seen, some of them containing autophagic structures. Interestingly, total axon count was also lower in GFP-LC3 mice compared to C576BL/6J controls (49,147 ± 6000 vs. 56,288 ± 7219 axons, *p* = 0.049, *n* = 10). RGC axons in GFP-LC3 mice showed early pathological features including focal degeneration with axonal swelling and enlargement^32^. More striking was the effect of the transgene in DBA/2J background. DBA/2J::GFP-LC3 mice exhibited a marked decrease in axon number compared to DBA/2J and GFP-LC3 mice (22,100 ± 9421 vs. 39,956 ± 13,692 and 49,147 ± 6000, respectively; *p* < 0.001, *n* = 10). Electron micrographs showed prominent gliosis with extensive degeneration across the whole nerve. Such degeneration, observed in 100% of the transgenic mice, was accompanied by the remarkable high presence of autophagic figures (arrows).

## Discussion

Here, we have investigated autophagy in the iridocorneal angle region, RGC bodies, and ON axons in the spontaneous ocular hypertensive DBA/2J mouse model of glaucoma. This is the first and only comprehensive study investigating the occurrence of autophagy in all the ocular tissues involved in the pathogenesis of hypertensive glaucoma.

As briefly mentioned in the Introduction, the role of autophagy in glaucoma has been the object of several studies (reviewed in refs.^13^^,^^15^). However, most of them have focused on RGC death, with hardly no documentation on axonal degeneration. Also, with the exception of in vitro work by our group, autophagy in ocular hypertension has not been extensively addressed in the literature. More worrisome, results obtained among the different reports are conflicting. The reasons underlying such high discrepancy are not fully understood, but it can be partly attributed to the use of different experimental models and/or analysis at different time-points. All of these studies were conducted in inducible experimental models of glaucoma, either by direct traumatic injury in the ON (ON transection, ON crush), acute IOP elevation (>110 mmHg, retinal ischemia/reperfusion model), or chronic IOP elevation (episcleral vein cauterization). Advantages here are the use of a more physiological model of hypertensive glaucoma, DBA/2J, highly resembling to human pigmentary glaucoma; and the generation of DBA/2J::GFP-LC3, expressing the autophagosome marker LC3 fused to the GFP reporter gene^31^.

Interestingly, the expression of the GFP-LC3 transgene did not have any effect in IOP levels in the C57BL/6J strain; however, it did in the DBA/2J background. DBA/2J::GFP-LC3 mice showed significantly higher cumulative IOP and developed elevated IOP around 1 month earlier, compared to the DBA/2J controls. Why GFP-LC3 expression leads to higher elevation in IOP in DBA/2J but not in C57BL/6J mice is currently not known. While no apparent morphological changes were observed in the DBA/2J::GFP-LC3 mice, ultrastructural analysis revealed the higher presence (in size and number) of autophagic digestive bodies within TM cells in the hypertensive transgene mice, which were just occasionally found in DBA/2J, GFP-LC3, and C57BL/6J mice. These data indicated alterations in basal autophagy in DBA/2J::LC3 mice.

Accurate quantification of autophagy in the outflow pathway tissue presented, however, two different challenges. First, it is not technically feasible to dissect the murine TM without contamination from adjacent tissues (cornea, iris). Although we acknowledge that data obtained in WB analysis from dissected iridocorneal tissue are not specific to the TM, it entirely correlated with that observed by immunostaining in the TM/SC region. The second challenge—also applicable to other tissues—comes from the dynamic nature of autophagy. Quickly after autophagy induction, new formed autophagosomes fuse with lysosomes to form autolysosomes, whereby material is degraded. In the autolysosomes, LC3-II is either degraded or recycled back to LC3-I. Therefore, steady-state LC3-II levels, as those evaluated in vivo, do not account for alterations in autophagic flux. Increased LC3-II levels or LC3 puncta can result from either autophagy activation or inhibition of the downstream degradative step^33^. Thus, although DBA/2J and DBA/2J::GFP-LC3 mice showed increased amounts of autophagosome-associated LC3 (LC3-II, LC3 puncta) compared to C57BL/6J and GFP-LC3 mice, it could not automatically be translated into higher autophagic activity. DBA/2J and DBA/2J::GFP-LC3 mice also demonstrated increased p62 and decreased LAMP1 protein levels. p62 is an autophagy receptor that is normally degraded by lysosomal proteases through the interaction with LC3-II, and accumulates when autophagy is inhibited, thus serving as a marker for autophagy flux. LAMP1 is a lysosomal-associated membrane protein commonly used to evaluate lysosomal content. The elevated levels of both LC3-II and p62 together with the decreased lysosomal content strongly suggests diminished autophagic flux in the outflow pathway region of DBA/2J hypertensive mice compared to age-matched C57BL/6J.

Diminished autophagic flux can result from either failure of autophagosomes to fuse with lysosomes, or from impaired degradative capacity within autolysosomes. Regarding the first possibility, similar to the findings here, a previous work by our lab reported higher LC3-II levels and the presence of autophagic vacuoles in outflow pathway cells in response to high pressure^34^. Although autophagic flux was not evaluated in situ, we confirmed both activation of autophagy and proper maturation of autophagosomes into autolysosomes in TM cells following application of mechanical stretch in vitro. Thus, cellular stretch associated to high IOP does not seem to affect per se autolysosome formation, at least at short-term. Alternatively, continuous autophagy activation with chronic IOP elevation or the presence of phagocytosed pigment particles can potentially saturate the degradative capacity in autolysosomes, as we previously described in aging TM cells, in which basification of the lysosomal compartment was associated with decreased autophagic flux^35^. Supporting this, TM cells phagocytically challenged to non-degradable particles showed higher lysosomal enzymes activities at day 2, that decreased afterwards to return to control values^36^. Ultrastructural images showed numerous pigment-loaded phagosomes in the outflow pathway cells of DBA/2J and DBA/2J::GFP-LC3 mice. However, the lower lysosomal content in the hypertensive mice is intriguing. In order to be effective, activation of autophagy must occur together with lysosomal biogenesis, so lysosomes are ready available to fuse with autophagosomes. This is transcriptionally regulated by the transcription factor TFEB^37^ and lysosomal efflux permeases, which export breakdown degradation products to the cytosol^38,39^. The fact that DBA/2J and DBA/2J::GFP-LC3 displayed decreased LAMP1 levels in the iridocorneal angle region could indicate uncoupling between autophagy induction and lysosomal degradation.

Evaluation of autophagy in RGC bodies revealed an overall decrease in the protein levels of LC3, p62, and LAMP1 in DBA/2J mice. Basal levels of autophagy in Brn3a-positive cells were observed in whole retinal flat mounts of DBA/2J::GFP-LC3 mice, but they did not differ from those observed in GFP-LC3 controls. These results, suggesting decreased autophagy in the RGCs of DBA/2J mice, somehow contradict previous studies in the literature using other experimental models. We should take into account, though, that our analysis was done in 12-month-old mice, 3–4 months after the onset of IOP elevation, whereas in the other studies autophagy was evaluated soon after IOP was experimentally raised. It is possible that cells in which autophagy was initially activated in our DBA/2J mice have already died. A more extensive analysis at different time-points will be necessary to evaluate this possibility.

Interestingly, expression of the GFP-LC3 transgene caused higher RGC degeneration in the ocular hypertensive, but not in control mice. More intriguingly is the apparent neurotoxic effect of GFP-LC3 expression in the ON of transgenic mice. GFP-LC3 mice displayed a 15% decrease in total ON axon number. Morphologically, axons looked stressed and showed signs of early damage including focal degeneration, swelling, and enlargement^32^. Such neurotoxic effect of GFP-LC3 dramatically intensified in the DBA/2J strain, with almost 50% of ON axon loss in DBA/2J::GFP-LC3 mice, accompanied by extensive gliosis and degeneration. Intriguingly, the most prominent feature in the ON axons from DBA/2J::GFP-LC3 mice—in addition to degeneration—was the massive presence of autophagic vacuoles. A number of axons containing autophagic vacuoles were also observed in DBA/2J mice, but in much lower number. Accumulation of autophagic vacuoles in the ON in DBA/2J and in the hypertensive glaucoma rat model have been previously reported^15^. In this later, LC3-II and p62 levels were observed to increase soon after IOP elevation indicating altered autophagic flux, similar to our own findings in the angle region. Both studies also described the accumulation of unhealthy mitochondria, which was attributed to loss of anterograde axonal transport in the DBA/2J mice and potential defect in mitophagy. However, as acknowledged, strong conclusions regarding the role of potential dysregulated autophagy, mitophagy, and neurodegeneration could not be reached due to the difficulties of assessing autophagic flux and mitophagy in vivo.

Two intriguing questions that remain unanswered from our studies are why transgene expression leads to ON axonal degeneration in C57BL/6J mice first place, and why degeneration is aggravated in combination with elevated IOP in the chronic hypertensive mice. GFP-LC3 mice have been widely used for the investigation of autophagy in vivo and so far, no abnormal phenotype has been described, not even when used as an experimental mouse model for other human diseases. In the original report, GFP-LC3 expression was found to be comparable to the level of the endogenous LC3 in the brain, but it was higher in other tissues, including the eye^31^. GFP-LC3 overexpression was reported to not affect endogenous autophagic activity, but autophagy activity triggered by exogenous LC3 was not evaluated. As mentioned earlier, our laboratory has shown activation of autophagy in response to high pressure^34^. It is plausible then that chronic IOP elevation in combination with greater LC3 availability (through GFP-LC3 overexpression) could lead to higher autophagy activity, resulting in cell death. That would explain the higher presence of autophagic vacuoles in the angle region and degenerating ON in DBA/2J::GFP-LC3 mice. Whether autophagy is directly activated by elevated IOP or indirectly through another mechanism or pathway triggered by elevated IOP cannot be inferred from our studies. Similarly, we cannot conclude whether the accumulation of autophagic bodies represents “autophagic cell death” or “cell death with autophagy” (attempt of the cell to induce autophagy as a survival pathway). Regardless, our data suggest ON axonal degeneration with dysregulation of autophagy.

In summary, we have described here for the first time impaired autophagic flux in the angle region of DBA/2J mice and ON axonal degeneration with dysregulation of autophagy in our DBA/2J::GFP-LC3 transgenic animal model, suggesting over-activation of autophagy as a potential cellular mechanism leading to ON degeneration in the chronic hypertensive DBA/2J mice. We believe DBA/2J::GFP-LC3 transgenic mice constitute an excellent tool to investigate the role of autophagy and the molecular pathways underlying neurodegeneration in glaucoma. We are currently generating in our lab additional tools to further and more precisely evaluate the contribution of autophagy to neurodegeneration in glaucoma.

## Materials and methods

### Animal husbandry, genotyping and tissue collection

Transgenic GFP-LC3 mice were obtained by rederivation of GFP-LC3#53 frozen embryos (RBRC00806,RIKEN BRC, Japan) in C57BL/6J using the DLAR Rederivation Core Facility services at Duke University. PCR-based genotyping of digested tail genomic DNA was performed under standard conditions using the following primers: GFP-LC3F1: ATAACTTGCTGGCCTTTCCACT; GFP-LC3R2:CGGGCCATTTACCGTAAGTTAT; GFP-LC3R3: GCAGCTCATTGCTGTTCCTCAA. Zygosity was determined based on the amplification of a wildtype 350 bp product (primers 1 and 3) or a 250-bp transgenic product (primers 1 and 2). GFP-LC3 mice were maintained and bred as heterozygous. Homozygous and littermate wildtypes were used for experimental purposes. To generate DBA/2J::GFP-LC3 mice, GFP-LC3 mice were bred with DBA/2J (Jackson Laboratories). Progeny was backcrossed for ten generations into the DBA/2J background. The presence of the Tyrp1^b^ allele was confirmed by assaying a polymorphism creating a *TaqI* restriction-enzyme site in exon 4 within PCR products amplified from genomic DNA (TyrpF: CAGGAGCCTTCTTTCTCCCT; TyrpR: AAAGTGTCCCAGGGTATCG). The presence of the Gpnmb^R150X^ mutation was confirmed by assaying the *PvuII* restriction-enzyme site created by the mutation (GpnmbF: CTACAACTGGACTGCAGGGG; GpnmbR: AGCTCCATTTCTTCCATCCA). Animals were maintained under a 12-hour light/dark cycle, fed a standard mouse diet, and provided with water ad libitum. Animal euthanasia was performed via CO_2_ asphyxiation, followed by bilateral thoracotomy, prior to immediate eye enucleation. Enucleated eyes were perfused with either 4% paraformaldehyde (PFA) for immunofluorescence or 2% glutaraldehyde/0.1 M cacodylate buffer for electron microscopy. Eyes were perfused by puncturing the eye at two opposing points just below the equatorial midline of the eye and infusing the eye with fixative very gradually so as not to perturb tissue structures. Tissues destined for WB or qPCR were immediately dissected and either flash frozen on dry ice or placed in RNAlater (Qiagen) on wet ice, respectively. All procedures were reviewed and approved by the Institutional Animal Care and Use Committee of Duke University and were performed in accordance with the ARVO Statement for the Use of Animals in Ophthalmic and Vision Research and the National Institutes of Health *Guide for the Care and Use of Laboratory Animals*. A total of 21 DBA/2J::GFP-LC3, 17 DBA/2J, ten C57BL/6J, and ten GFP-LC3 mice were aged up to 12 months and used in this study.

### IOP measurements

Diurnal IOP was measured on a monthly basis using a Tonolab rebound tonometer held approximately 1–4 mm from the top of the probe to the cornea. The mice were sedated with an intraperitoneal injection of Ketamine (100 mg/kg) and Xylazine (5–10 mg/kg). Immediately following onset of anesthesia, Proparacaine HCl (0.5%) drops were applied to the eyes to prevent corneal reflex in response to probe stimulation. A series of six measurements were collected per eye, and these values were averaged to produce a single IOP value per eye for each measurement session. Deltaphase isothermal pads (Braintee Scientific, Inc.) were used to maintain body temperature at 38–40° until recovery from anesthesia, during which drops of 1× PBS were applied to the eyes to prevent drying out of the cornea. Cumulative IOP (mmHg/month) was calculated.

### Immunofluorescence on frozen tissue sections

Tissue samples were fixed in 4% paraformaldehyde (PFA) for 24 h and subsequently transferred to a 1% PFA solution for long-term storage. Prior to embedding, tissue samples were subjected to an increasing sucrose gradient (10%, 20%, 30% sucrose in 1× PBS) and embedded in O.C.T. compound (Tissue-Tek), rapidly frozen via methanol chilled by dry ice. Embedded tissue was sectioned on a cryostat, collected on gelatinized slides, and stored at −80 °C until stained. Before immunostaining, slides were thawed at 42 °C for 10 min and washed in 1× PBS to remove excess O.C.T. Samples were permeabilized by incubating in 0.5% Triton-X/PBS at room temperature for 10 min and washed in PBS. Non-specific sites were blocked by incubation for 30 min at room temperature in blocking solution (2% BSA/5% normal goat serum/0.1% Triton-X/1× PBS). Samples were then incubated overnight at 4° with primary antibodies diluted at their respective ratios in blocking solution, washed with 1× PBS and incubated in Alexa-Fluor Goat anti-Rabbit 568 (Life Technologies) diluted 1:1000 in serum-free blocking solution for 1 h at room temperature. Nuclei were counterstained with DAPI (1:1000 in PBS) and sections were mounted in aqueous mounting media (Fluormount G; Electron Microscopy Sciences) and coverslipped. Images were collected using a Nikon TE2000 confocal microscope using a 63× objective. All tissues were immunostained at the same time and images were captured the same day under the same laser settings to avoid interexperimental variability. Standardized relative quantification of immunofluorescence staining was calculated using Image J as described in ref.^40^. The following antibodies were used in this study: anti-LC3 (MBL International), anti-p62 (P0067, Sigma), and anti-LAMP1 (ab24170, Abcam).

### Whole retinal flat mounts and RGC quantification

Bisected retinas fixed in 4% PFA were washed three times for ten minutes in 0.5% Triton-X/1× PBS at room temperature, and then permeabilized overnight in 2% Triton-X/1× PBS at 4 °C with agitation. Retinas were subsequently incubated for 3 days at 4 °C with anti-Brn3a (Santa Cruz Biotechnology) diluted 1:750 in 2% Triton-X/2% normal donkey serum/1× PBS. Following washing in 2% Triton-X/1× PBS and 0.5% Triton-X/1× PBS, samples were incubated at room temperature for 4 h in a 1:500 dilution of Alexa-Fluor donkey anti-goat 568 (Life Technologies) in 2% Triton-X/1× PBS and counterstained in Hoechst (1:1000 in 0.5% Triton-X/1× PBS) for 30 min at room temperature. Samples were washed in 0.5% Triton-X/1× PBS, mounted in aqueous mounting media (Fluormount G; Electron Microscopy Sciences), and coverslipped. Retinal flat mounts were imaged with a EZ-C1.3.10 Nikon TE2000 confocal microscope (40× lens) (D-ECLIPSE C1, Nikon). A series of four images were taken at approximately equidistant points along peripheral circumference of the retina, with a final image being taken proximal to the optic disc. The collected images were then quantified and averaged using a custom semi-automatic application developed for MATLAB which identified RGCs based on color, shape, and size.

### Electron microscopy

Whole eye cups were post-fixed for 24 h in 2% glutaraldehyde/0.1 M cacodylate buffer solution and then transferred to cold 3% glutaraldehyde/0.1 M cacodylate buffer for at least 2 h. Samples were then washed in cacodylate washing buffer and post-fixed for 1.5 h in 2% OsO4 in 0.1 M cacodylate buffer. Complete dehydration was achieved using an increasing ethanol gradient ending in two cycles of propylene oxide. Tissue was infiltrated by immersion in a 1:1 mixture of propylene oxide and Epon 812 resin under a vacuum for 4–10 h. Labeled molds were then heated at 68 °F for at least 8 h. Sections were cut at 65 nm thick using a Leica EM CU7 and contrast stained with 2% uranyl acetate/4% lead citrate solution. Ultrathin sections were visualized on a JEM-1400 transmission electron microscope (JEOL) using an ORIUS (1000) CCD camera.

### ON axon count

ONs were fixed in 4% paraformaldehyde, 1% glutaraldehyde, and 0.12 M phosphate buffer overnight. The samples were processed and embedded in the Embed-812 resin mixture. Blocks were sectioned on an ultramicrotome (LKB Ultratome V; Leica) using a glass knife. Cross-sections were stained with 1% toluidine blue and coverslipped. Axon counts were obtained using the AxioVision imaging system (Zeiss). Image analysis consisted of RGB thresholding, followed by size and form factor exclusions. Approximately 40% of the total cross-sectional area for each ON was counted, and the results were extrapolated to the entire nerve for each mouse.

### Protein whole tissue lysate preparation western blots

Dissected tissue was homogenized by manual grinding in cold RIPA buffer containing protease and phosphatase inhibitor cocktails (Thermo Scientific) and molecular grinding resin (G Biosciences). Lysates were subjected to three freeze/thaw cycles and clarified by centrifugation at 12,000 × *g* for 30 min at 4 °C. Protein concentration was determined with a protein assay kit (Micro BCA, Thermo Scientific). Protein lysates (5–10 μg) were separated by polyacrylamide SDS-PAGE gels [15% polyacrylamide for LC3 detection (NB100–2331 from Novus Biologicals), 7% polyacrylamide for LAMP1 (ab24170 from Abcam) and p62 (P0067, Sigma detection)] and transferred to PVDF membranes (Bio-Rad). Membranes were blocked with 5% non-fat dry milk in 0.1% Tween-20/TBS and incubated overnight with primary antibodies. The bands were detected by incubation with a secondary antibody conjugated to horseradish peroxidase and chemiluminescence substrate (ECL; GE Healthcare and ECL2, Thermo Scientific). Blots were scanned and analyzed by densitometry using Image J. β-Actin (sc-69879) was used for loading control.

### Statistical analysis

All statistical analyses were performed using GraphPad Prism software. Data are presented as mean values ± SD using the Tukey post-hoc test. *P* < 0.05 was considered statistically significant.

## Electronic supplementary material

MOrphologoy angle and retina structure in transgenic mice
