# Supplementary material for: Contribution of autophagy to ocular hypertension and neurodegeneration in the DBA/2J spontaneous glaucoma mouse model
Source: Cell Death Discov. 2018 Jul 17;4:75. doi: 10.1038/s41420-018-0077-y (PMC6127277; doi:10.1038/s41420-018-0077-y)

**SUPPLEMENTARY INFORMATION**

Cross-sectional thick sections of the angle region (A) and retina tissue (B) of control and experimental mice. TM: trabecular meshwork; SC: Schlemm’s canal; GCL: ganglion cell layer; IPL: inner plexiform layer; INL: inner nuclear layer; OPL: outer plexiform layer; ONL: outer nuclear layer


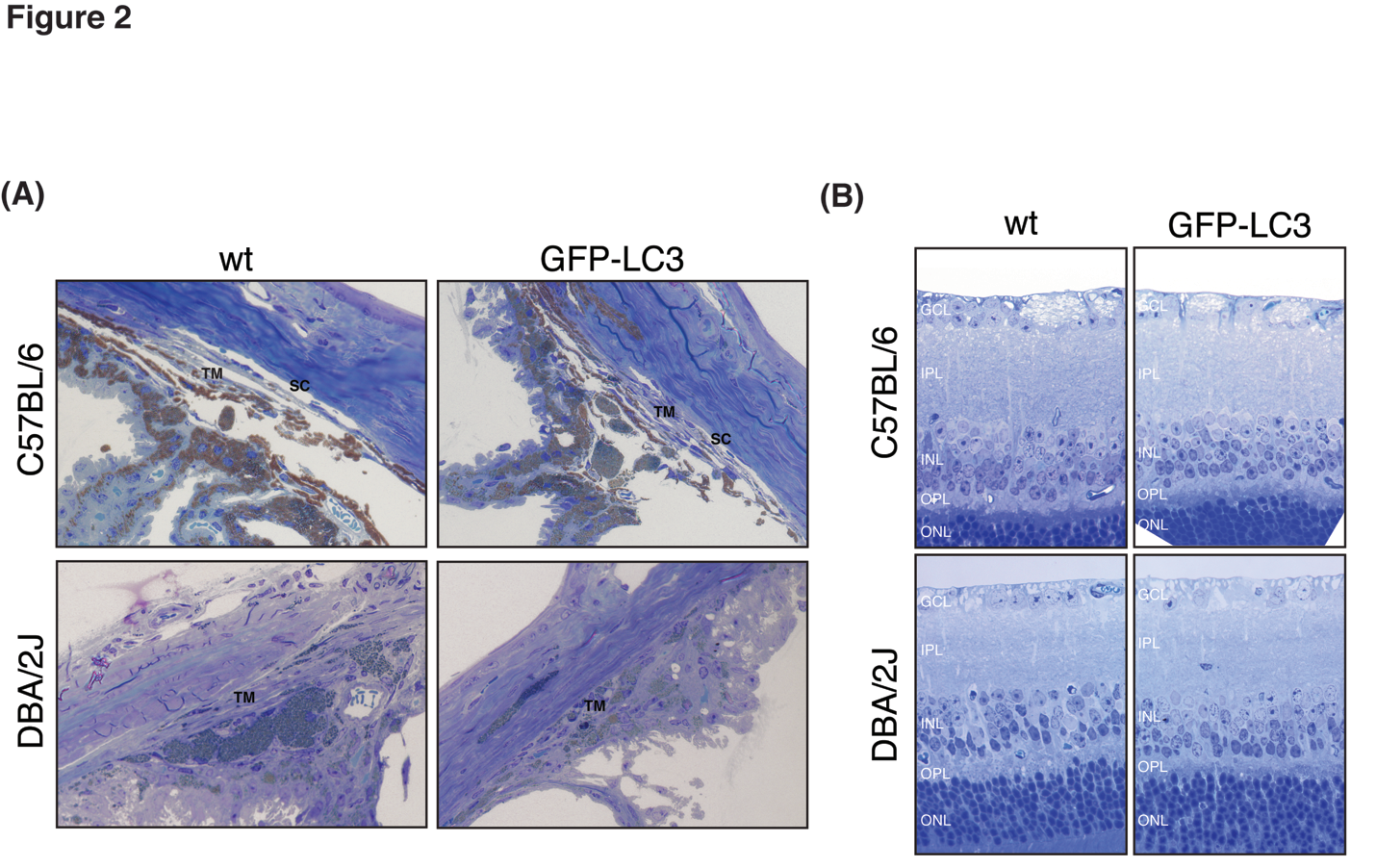

Supplement: Supplementary file 1 — MOrphologoy angle and retina structure in transgenic mice [file 41420_2018_77_MOESM1_ESM.docx]
